# Commercial microbial polysaccharides: An annotated selection of World Wide Web sites relevant to the topics in Microbial Biotechnology

**DOI:** 10.1111/1751-7915.12053

**Published:** 2013-04-15

**Authors:** Lawrence P Wackett

**Affiliations:** 1 Department of Biochemistry, Molecular Biology and Biophysics, BioTechnology Institute, University of MinnesotaSt Paul, MN, 55108, USA

## Microbial polysaccharides

http://www.sgm.ac.uk/pubs/micro_today/pdf/050204.pdf

This review article provides a good overview of bacterial exopolysaccharides and their commercial significance.

## Microbial polysaccharides: Review

http://www.nottingham.ac.uk/ncmh/harding_pdfs/Paper322.pdf

This review gives a comprehensive description of the structure, function and diverse applications of bacterial polysaccharides.

## Dextran and related polysaccharides

http://www.sigmaaldrich.com/technical-documents/articles/biofiles/dextran-and-related.html

Dextrans are among the oldest known bacterial polysaccharide products and are widely used in foods, cosmetics and biotechnology.

## Subterranean permeability modification using polysaccharides

http://www.google.com/patents/US4941533

This patent describes a bacterial exopolysaccharide with usefulness in enhanced oil recovery.

## Bacterial polysaccharide database

http://sydney.edu.au/science/molecular_bioscience/BPGD/default.htm

This database provides some information on genes and pathways involved in the biosynthesis of bacterial polysaccharides.

## Gellan

http://www.lsbu.ac.uk/water/hygellan.html

Gellan is an exopolysaccharide produced by aerobic fermentative growth of *Sphingomonas elodea*.

## Gellan gum

http://en.wikipedia.org/wiki/Gellan_gum

Gellan gum is a water-soluble polysaccharide used as a microbiological gelling agent and in foods. This website gives the name of the producing organism as a *Pseudomonas*, but that organism was more recently reclassified as a *Sphingomonas* strain.

## Guar gum replacements

http://www.gumtech.com/products/GuarReplace.php

Guar gum is a plant polysaccharide that is used in foods and, most recently, in hydraulic fracturing to produce oil and gas. There is an ongoing search to find replacements due to the huge volumes required for the latter application.

## Xanthan gum: Wikipedia

http://en.wikipedia.org/wiki/Xanthan_gum

Xanthan gum is a polysaccharide secreted by the bacterium *Xanthomonas campestris* that is widely used in the food industry, as described in this Wikipedia entry.

## Levan

http://www.polysaccharides.us/aboutlevan_background.php

Levan is a polysaccharide made of fructose that is unusual by packing into a highly stable spherical structure. This website describes many of the properties and used of the polymer.

## Aureobasidium pullulans

http://www.mycology.adelaide.edu.au/Fungal_Descriptions/Hyphomycetes_(dematiaceous)/Aureobasidium/

This fungus produces the exopolymer pullulan.

## Spirulan

http://www.elicityl-oligotech.com/?fond=rubrique0026;id_rubrique=30

Spirulan, produced by certain strains if filamentous cyanobacteria, is an acidic, sulfated polysaccharide.

## Recombinant hyluronan

http://www.biopharma.novozymes.com/en/products/hyaluronic-acid/Pages/default.aspx

Hyaluronan is an important biomedical polymer. Its sources have been limited and its recombinant production is an important development.

